# Penetrance of the *TP53* R337H Mutation and Pediatric Adrenocortical Carcinoma Incidence Associated with Environmental Influences in a 12-Year Observational Cohort in Southern Brazil

**DOI:** 10.3390/cancers11111804

**Published:** 2019-11-16

**Authors:** Tatiana E. J. Costa, Viviane K. Q. Gerber, Humberto C. Ibañez, Viviane S. Melanda, Ivy Z. S. Parise, Flora M. Watanabe, Mara A. D. Pianovski, Carmem M. C. M. Fiori, Ana L. M. R. Fabro, Denise B. da Silva, Diancarlos P. Andrade, Heloisa Komechen, Monalisa C. Mendes, Edna Carboni, Ana Paula Kuczynski, Emanuelle N. Souza, Mariana M. Paraizo, Marilea V. C. Ibañez, Laura M. Castilho, Amanda F. Cruz, Thuila F. da Maia, Cleber Machado-Souza, Roberto Rosati, Claudia S. Oliveira, Guilherme A. Parise, Jaqueline D. C. Passos, José R. S. Barbosa, Mirna M. O. Figueiredo, Leniza Lima, Tiago Tormen, Cesar C. Sabbaga, Sylvio G. A. Ávilla, Leila Grisa, Airton Aranha, Karina C. F. Tosin, Karin R. P. Ogradowski, Geneci Lima, Edith F. Legal, Tania H. Anegawa, Tânia L. Mazzuco, André L. Grion, José H. G. Balbinotti, Karin L. Dammski, Rosiane G. Melo, Nilton Kiesel Filho, Gislaine Custódio, Bonald C. Figueiredo

**Affiliations:** 1 Instituto de Pesquisa Pelé Pequeno Príncipe, Av. Silva Jardim, 1632, Curitiba 80250-060, PR, Brazil; 2 Faculdades Pequeno Príncipe, Av. Iguaçu, 333, Rebouças, Curitiba 80230-020, Brazil; 3 Hospital Infantil Joana de Gusmão, R. Rui Barbosa, 152, Agronômica, Florianópolis 88025-301, SC, Brazil; 4 Departamento de Enfermagem, Universidade Estadual do Centro-Oeste, Rua Simeão Varela de Sá, 03, Vila Carli, Guarapuava 85040-080, PR, Brazil; 5 Secretaria do Estado da Saúde do Paraná, R. Piquiri, 170, Rebouças, Curitiba 80230-140, PR, Brazil; 6 Hospital Pequeno Príncipe, Rua Desembargador Motta, 1070, Água Verde, Curitiba 80250-060, PR, Brazil; 7 Oncologia Pediátrica, Hospital Erasto Gaertner, R. 201, Jardim das Américas, Curitiba 81520-060, PR, Brazil; 8 Hospital do Câncer, UOPECCAN, R. Itaquatiaras, 769, Santo Onofre, Cascavel 85806-300, PR, Brazil; 9 Centro de Genética Molecular e Pesquisa do Câncer em Crianças (CEGEMPAC-APACN), Av. Agostinho Leão Junior, 400, Alto da Glória, Curitiba 80030-110, PR, Brazil; 10 Oncologia Pediátrica, Hospital de Clínicas da Universidade Federal do Paraná, R. Gen. Carneiro, 181, Alto da Glória, Curitiba 80060-900, PR, Brazil; 11 Oncologia Pediátrica, Universidade Estadual de Londrina, Rodovia Celso Garcia Cid-PR 445 Km 380, Campus Universitário, Londrina 86057-970, PR, Brazil; 12 Oncologia Pediátrica, Hospital do Câncer de Londrina, Rua Lucilla Ballalai, 212, Jardim Petrópolis, Londrina 86015-520, PR, Brazil; 13 Divisão de Endocrinologia, Departamento de Clínica Médica, Universidade Estadual de Londrina, Rua Robert Koch, 60, Vila Operária, Londrina 86038-350, PR, Brazil; 14 Departamento de Saúde Coletiva, Universidade Federal do Paraná, R. Padre Camargo, 280, Alto da Glória, Curitiba 80060-240, PR, Brazil

**Keywords:** R337H, *TP53*, Li–Fraumeni syndrome, adrenocortical carcinoma, children, environmental modifiers

## Abstract

The *TP53* R337H mutation is associated with increased incidence of pediatric adrenocortical tumor (ACT). The different environmental conditions where R337H carriers live have not been systematically analyzed. Here, the R337H frequencies, ACT incidences, and R337H penetrance for ACT were calculated using the 2006 cohort with 4165 R337H carriers living in Paraná state (PR) subregions. The effectiveness of a second surveillance for R337H probands selected from 42,438 tested newborns in PR (2016 cohort) was tested to detect early stage I tumor among educated families without periodical exams. Estimation of R337H frequencies and ACT incidence in Santa Catarina state (SC) used data from 50,115 tested newborns without surveillance, ACT cases from a SC hospital, and a public cancer registry. R337H carrier frequencies in the population were 0.245% (SC) and 0.306% (PR), and 87% and 95% in ACTs, respectively. The ACT incidence was calculated as ~6.4/million children younger than 10 years per year in PR (95% CI: 5.28; 7.65) and 4.15/million in SC (CI 95%: 2.95; 5.67). The ACT penetrance in PR for probands followed from birth to 12 years was 3.9%. R337H carriers living in an agricultural subregion (C1) had a lower risk of developing pediatric ACT than those living in industrial and large urban subregion (relative risk = 2.4). One small ACT (21g) without recurrence (1/112) was detected by the parents in the 2016 cohort. ACT incidence follows R337H frequency in each population, but remarkably environmental factors modify these rates.

## 1. Introduction

Pediatric malignancies represent about 1.5% of all human malignancies [1]; data from the American National Cancer Institute suggest that pediatric adrenocortical tumors (ACT) are very rare, representing about 0.2% of all childhood malignancies [2]. In Paraná state (PR) in Southern Brazil, however, the frequency of ACT is at least 15 times greater (per million children) than in Los Angeles (0.4/million) and 60 times greater than in Hong Kong or Bombay (0.1/million) [3,4,5]. Germline mutations in the *TP53* gene are associated with Li–Fraumeni syndrome (LFS) and are responsible for the majority of ACT cases in young children worldwide, which merits genotyping and counseling, including for low-penetrance *TP53* mutation carriers [6,7]. Although approximately 80% of pathogenic *TP53* mutations occur within the DNA binding domain (DBD) [8], *TP53* R337H, which is the single most commonly found germline mutation [9], is located in the tetramerization domain in exon 10 (p.Arg337His, c.1010G > A). *TP53* R337H is a founder mutation [10], and its increasing frequency in the population is facilitated by its low penetrance, with a relatively low cancer risk during the reproductive years. However, it is not clear how this mutation accumulated only in Southern Brazil from a Caucasian/Portuguese–Iberian common ancestor [11]. While R337H occurs in 1:370 births in Paraná state [3], other *TP53* germline mutations are thought to occur at a frequency of between 1:5000 and 1:20,000 births [7,12] in other countries. These conclusions were based on mutations found in patients with early onset breast cancer unselected for family history (2–3%) [12] or any type of cancer family history (17.3%) [7]. A lack of accuracy in these frequencies could be attributed to selection criteria [13], inclusion of somatic and germline mutations, and differences in haplotype penetrance. Predisposition of R337H carriers and non-carriers to ACT has an early peak in the first three years after birth (virilization syndrome more common than Cushing syndrome), decreases with age toward a second phenotype (Cushing syndrome > single virilizing syndrome) in the second decade, and is associated with a poorer ACT prognosis in adulthood [13,14,15]. The elevated susceptibility of the adrenal cortex to ACT during the first postnatal years may be related to fetal zone instability around birth, and the expected incidence of ACT is proportional to the number of children born with the germline *TP53* R337H mutation [13]. In addition, a minor contribution to tumorigenesis is attributed to genetic and epigenetic alterations affecting chromosome 11p15 [16,17], as well as other unknown constitutional predispositions or environmental factors. For example, the most potent toxic compounds impacting the adrenal cortex and ACT development are the organochlorines (OCs) dichlorodiphenyltrichloroethane (*p*,*p*′-DDT) and its main derivatives dichlorodiphenyldichloroethylene (*p*,*p*′-DDE) and dichlorodiphenyldichloroethane (*p*,*p*′-DDD), which were detected at high levels in some regions of Paraná state [18]. 

A small hospital-based cohort study (~1000 participants) estimated that the penetrance for ACT is approximately 10% [19]. However, in a surveillance control and larger normal population-based and prospective cohort of R337H-carrier newborns, the penetrance was 3.4% for children younger than five years of age [3]. These authors found a 1:15 (adult:pediatric) ACT frequency, which is in line with the R337H carrier rate previously reported [20]. Testing newborns for R337H, combined with specific surveillance for ACT, has improved the early preclinical diagnosis of ACT and raised the cure rate [3]. However, higher-penetrance *TP53* mutations merit extended surveillance for children and adults with diagnoses of early onset tumors [21]. Phenotypic heterogeneity among R337H carriers may indicate the involvement of other genetic variants that are able to modulate ACT development [22]. However, the estimation of ACT risk is partially hampered by a lack of in-depth knowledge of environmental influences on R337H carriers. 

The aims of this study were (1) to determine the frequency of the R337H carrier in newborns and the overall ACT incidence among children of Paraná (PR) and Santa Catarina (SC) states; (2) to compare the ACT incidence and estimate relative risk (RR) among three PR subregions with distinct environmental conditions (C1 with very strong agriculture production; C2 with moderate agriculture and mild industry; and C3 with strong industry activity and mild agriculture production); and (3) to test a cost-effective surveillance method to detect early stage ACT among R337H-carrier children from PR. To test the hypothesis that R337H frequency and different environmental factors influence the incidence of pediatric ACT, we evaluated differences in the cumulative ACT incidence according to the total number of R337H carriers in our cohort (2006-C) living in C1, C2, and C3. This hypothesis was validated using a public ACT registry database [23] that matched with ACT hospital registries. The second neonatal R337H screening in PR empowered estimates of R337H frequencies and established a second cohort of R337H-carrier newborns efficiently monitored without periodical exams. We performed the first R337H neonatal screening in SC without any surveillance (as approved by the Ethics Committee just to confirm the epidemiological problem), and the obtained frequency of R337H in pediatric ACT from SC to compare with PR’s status.

## 2. Results

### 2.1. TP53 R337H Frequency in the Southern Brazilian Population

**Figure 1:**
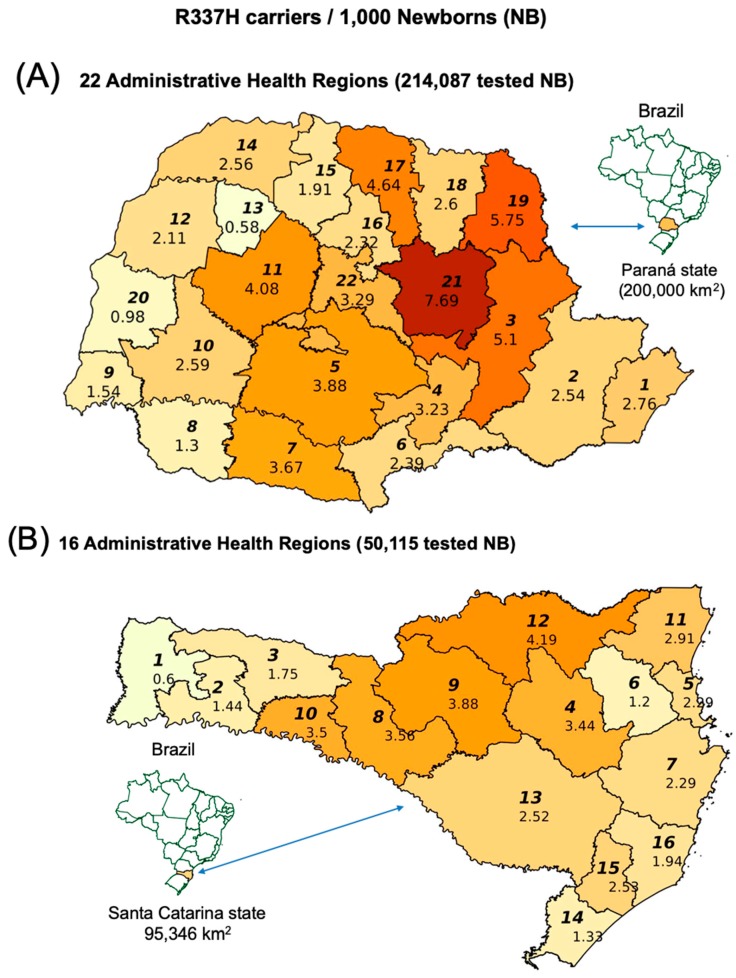
(**A**) R337H frequencies among newborns from 22 Paraná Administrative Health Regions (AHRs) and (**B**) from 16 Santa Catarina AHRs (stored blood collected in 2013–2014). AHRs are represented by bold italic numbers.

Geographic differences in the R337H frequencies in newborns (NBs) from all Administrative Health Regions (AHR) of two southern Brazilian states are shown in Figure 1. More accurate frequencies in the 22 Paraná AHRs were estimated by adding 42,438 new tests to the previously tested 171,649 newborns [3], for a total of 214,087 tested newborns. The total number of births per year in PR is ~155,000. The updated average population R337H frequency was 0.306%, ranging from 0.058% to 0.769% in these 22 AHRs (Figure 1A). We performed 50,115 R337H tests on dried blood stored on Guthrie cards in the Santa Catarina (SC) state repository (from newborns born in 2013 and 2014) in each of the 16 AHRs, corresponding to over 50% of the total number of births per year (~92,000 births). The average R337H frequency in the SC population was 0.249%, ranging from 0.06% to 0.49% (>3000 tested newborns/AHR). The highest frequencies were found in the central AHRs closest to PR (Figure 1B).

### 2.2. TP53 R337H Frequency in Adrenocortical Tumors from Santa Catarina

From 2000 to 2018, 35 ACT patients younger than 10 years and three between 10 and 14 years of age were admitted at the Joana Gusmão Children’s Hospital (Florianópolis, SC capital), which is responsible for the care of more than 60% of all cancer patients in SC. Thirty-three patients (86.8%) were heterozygous for the R337H haplotype, and five were wild type.

### 2.3. Cumulative Incidence of Pediatric ACT in the PR 2006-C Cohort According to Subregion

Three distinct subregions were previously identified: C1 with strong agriculture; C2 with moderate agriculture and mild industry; and C3 with strong industry activity and mild agriculture production. Higher levels of DDT/DDD/DDE were detected in C1, intermediary levels in C2, and lower levels in C3. A small subregion of the PR territory without geochemistry analyses was classified as C0 [18]. Retrospective (N = 51) and prospective (N = 26) instances of pediatric adrenocortical tumors were identified in 409 families in the 2006-C cohort. Fifty-three new families (previously non-adherent) decided to participate five to seven years after their index cases (NBs) were identified as positive for R337H on the neonatal screening. These 77 ACT cases were identified in 66 families with one or more cases among children younger than 10 years (N = 76), plus one at 14 years (N = 1) and three cases among adult R337H carriers since 2006. The cumulative incidence for childhood ACT among all 4162 carriers was 0.95% in C1, 1.83% in C2, and 2.47% in C3. Remarkably, the relative risk (RR) to develop ACT was 2.41 (1.07; 5.39) in C3 (*p* < 0.031) as compared with C1 (Table 1). The number of ACT cases divided by the number of carriers in each subregion for the 2006 cohort identified one ACT for each 105 R337H carriers in C1, in contrast to the 1:44 proportion in C3, suggesting that it is easier to develop ACT in C3 and/or the risk is lowered in C1. This conclusion was further validated using the public ACT registry of patients admitted to public hospitals (patients without medical insurance).

### 2.4. Pediatric ACT Incidence Rates in Paraná and Santa Catarina

The ACT incidence estimated for PR was 6.38 per million children per year until nine years of age (95% CI: 5.28; 7.65). The significant differences in the frequency of ACT development between C1 and C3 in the 2006-C cohort considering the number of ACT cases and the number of carriers in each subregion (Table 1) were further validated using data on ACT cases from the public cancer registry [23]. The number of identified patients under 10 years old in PR between 2007 and 2018 (N = 118) was used to calculate the ACT incidence. Differences in ACT incidence rate were calculated using the estimated population per year from 2007 to 2018 in PR and in each PR subregion (C0, C1, C2, and C3) (Table 2A). The incidence rate in C3 is 2.7 times (IRR) higher than in C1 (*p* = 0.0053, Bonferroni adjusted) (Table 2B), which validates our cohort result shown in Table 1. 

Dividing the number of ACT cases by the number of estimated R337H carriers in each subregion demonstrated that there is one ACT for every 93 R337H carriers in C1 and one ACT for every 26 carriers in C3 for children less than 10 years of age. These proportions are slightly different to the calculated proportions using the 2006-C cohort (1:44 and 1:103), because the database from the Brazilian Single Health System (DATASUS) registry (1) included a low percentage of ACT cases from other small public hospitals, (2) may have included non-R337H ACTs, and (3) may have excluded patients with private medical health insurance. Cancer patients with health insurance correspond to ~22% of all patients according to National Health Agency [24]. 

The calculated ACT incidence in SC (using 39 ACT cases from the public database), based on ACT cases identified from 2007 to 2017 [23], was 4.15 per million children per year in children younger than ten years (CI 95%: 2.95; 5.67). 

### 2.5. Linear Regression (LR) for R337H Frequencies: General Population versus ACT Patients

**Figure 2:**
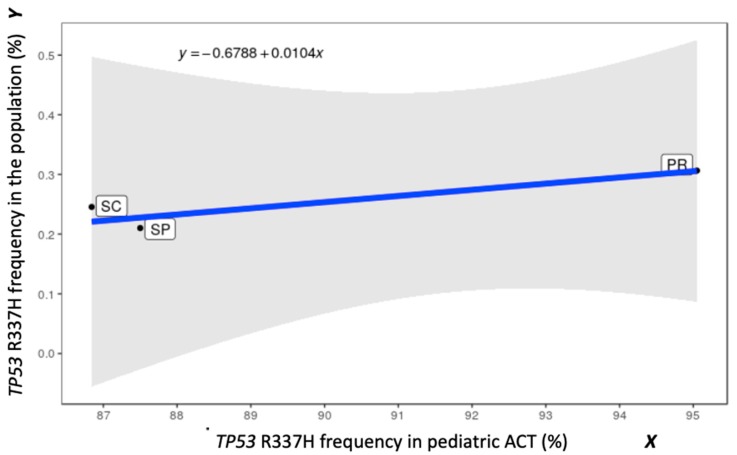
The R337H frequencies (population versus ACT) and the obtained linear regression (LR) based on the overall averages for each state (SP, Paraná state (PR), and Santa Catarina (SC)). The blue straight line was obtained by simple LR using the method of least squares weighted by the number of individuals tested in each state, assuming equal weights for the considered variables. The shaded area represents the 95% confidence interval of the line assuming the normality of the errors.

In addition to the neonatal screening in SC, we tested 35 pediatric ACT patients admitted to the main SC pediatric hospital (Joana Gusmão Pediatric Hospital at Florianópolis) from 2000 to 2018 and found 30 R337H positive and five R337H negative tumors. R337H frequencies reported in the population and in ACT by other studies in PR [3,9,25], and in SP [20,26,27,28], were also used to estimate the LR. The mean R337H carrier frequencies in the general population (Y) were 0.210% (SP) according to data by Caminha et al. [28], 0.245% (SC), and 0.306% (PR) (calculated in the present study) associated with 87%, 87%, and 95% R337H frequencies in pediatric ACTs, respectively. Paired coordinates of the average frequencies of R337H-positive ACT (X) and of R337H carriers in the general population (Y) from SP, PR, and SC states were used to detect the approximate LR (*Y* = −0.6788 + 0.0104*X*) (Figure 2). The limitations of this LR are predictable as the information is restricted to only three coordinates. In addition, the SP R337H population frequency data was restricted to one small region (metropolitan area of Campinas-SP). Consequently, inferences may also be prone to small distortions between estimates versus real measurements of both frequencies in unknown places, within or around these three states.

### 2.6. ACT Surveillance in Paraná: 2006-C and 2016-C Cohorts

Among the 42,438 tested NBs in PR (born between 2015 and 2018), 147 were heterozygous for R337H, but only 112 NBs and their families adhered to the observational trial studying a new surveillance design without periodic exams (2016-C). The parents and most relatives were counseled and received training about cancer manifestations as well as psychological support during three sessions that were three hours in length at two-month intervals after the birth of the proband (2016-C cohort). Specific exams were provided only to clarify suspicious clinical manifestations of ACT or other cancers spontaneously reported by the parents or during periodic inquiries by telephone. The cancer history was taken, and all available relatives from the carrier side were offered genetic and psychological counseling, testing, and periodic support (preclinical exams (PE)). This 2016-C (without PE) cohort was followed in parallel with the 2006-C cohort under full surveillance with medical, hormonal, and ultrasound exams at 3-, 6-, or 12-month intervals according to age [3]. Three new cases of pediatric ACT were identified among the 353 children in the 2006-C cohort followed since birth, with a mean follow-up time of almost 10 years (Table 3A). Only one ACT case (girl at the age of 9.4 months, presenting a very small ACT) was identified among the 112 R337H-carrier children of the 2016-C group, with a mean follow-up time of 2.7 years (Table 3B). Exclusion of PE associated with a small cost for educating the parents of the probands significantly reduced the costs of the surveillance.

### 2.7. Revised Estimate of R337H Penetrance in the Paraná 2006-C Cohort

Among all 353 R337H-carrier newborns followed since their births between 2006 and 2019, 14 developed ACT before turning 12 years old, including 11 probands previously reported before completing five years of age [3] and three more recent cases (Table 3A). The present median follow-up time for participants of all ages in the cohort until April 2019 was 9.84 years (range, 8.67–12.01 years). The probands continued under the same surveillance protocol with PE that previously detected a 3.4% penetrance for children under five years of age until 2012 [3]. ACT cases among siblings and other relatives were excluded from this analysis. The estimated 3.96% penetrance was further adjusted for the total children born per year in PR (~155,000, 2012 census) taking into account the previously identified R337H newborns (461 carriers/171,649 tested newborns). Thus, it is expected that approximately 416 newborns will be found to carry R337H every year. We conclude that approximately 16 children (from all socioeconomic backgrounds, including patients with private medical insurance) born every year carrying the R337H mutation will develop ACT before turning 12 years old. In addition, at least one or two non-carrier children may develop ACT among those born each year [3].

### 2.8. Age of Pediatric ACT Onset in the PR 2006-C Cohort According to Generation

**Figure 3:**
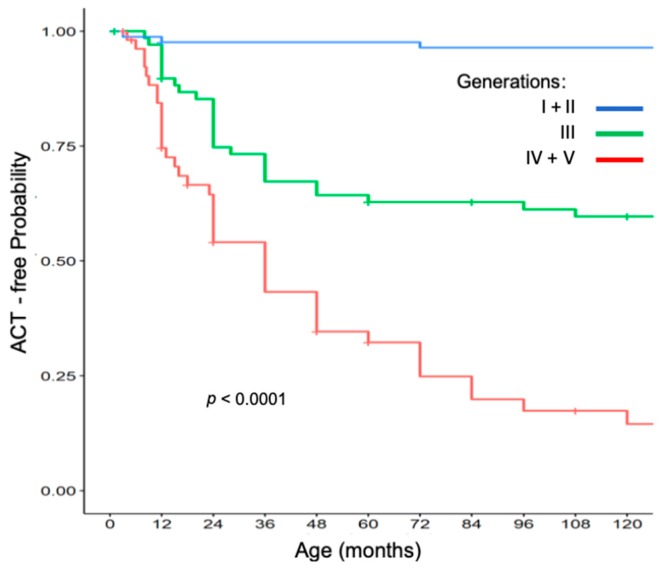
ACT-free probability in the 2006-C cohort.

Data from carriers from all families were subdivided according to the generation (Figure 3). The first two (I + II) and the last two generations (IV + V) of all families, from all subregions were combined to augment statistical power. Despite the fact that different environmental conditions could have opposite results, a striking earlier age of ACT onset was observed in the last generations (*p* = 0.0001). 

## 3. Discussion

Our results provided new insights into the heterogeneity of adrenal cortex tumor development in young children living in different subregions in southern Brazil. Data from the 2006 and 2016 cohorts revealed that *TP53* R337H is a low-penetrance mutation based on the observed average lifetime cancer risks, estimated as less than 40% in our 2006 cohort. We further confirmed the 2006 cohort findings [3] that *TP53* R337H is a low-penetrance mutation with a low lifetime cancer risk. Carriers may escape cancer, as the carrier side of a family demonstrates. On average, only 3.9 more cancer cases of all types occur in the carrier side of the family than in the non-R337H segregating side of the family, in contrast to the high penetrance and high lifetime cancer risk reported in classical LFS [29]. Despite the fact that the R337H mutation lies within the p53 dimerization domain which may affect tetrameric stability, R337H had wild-type p53 activity in in vitro assays, in contrast to the profound defects in DNA binding domain mutant reported in LFS [9]. The pH-dependent destabilization of p.R337H tetramer demonstrated in vitro [30] has not yet been validated in human cells. One interesting aspect of R337H carriers in our two cohorts is the variable frequency of pediatric ACT among different PR subregions. The 2016-C cohort was strategically selected to include more C1 municipalities in Paraná state, where only one 0.93-year old girl developed ACT in the first 30 months of follow-up of this 2016 cohort. This finding is in line with the detailed differences found between C1 and C3 in the long-term follow-up of the 2006 cohort, which was geographically classified in our recent environmental analysis [18]. 

The frequency of the germline R337H mutation among Paraná newborns [3] was refined in the present study after adding results from 42,438 newborns to obtain more accurate estimates. (A total of 214,087 tests or ~140% of the total number of births in one year were analyzed). The updated average frequency of R337H carriers in PR (0.306%) is the highest, followed by that in SC (0.245%), and SP (0.21%), which was partially consistent with the highest R337H frequencies estimated in ACTs for PR (95%), SP (87%), and SC (87%). It is also consistent with the highest ACT incidence in PR, with 6.38/million in children younger than 10 years admitted to public hospitals (95% CI: 5.28; 7.65). This rate is slightly different from that observed in our 2006 cohort, likely because patients with medical insurance are included in the cohorts. However, our data demonstrated that ACT incidence is not homogenous in PR, with the exception observed in PR subregion C1, which does not have the frequency of ACT cases compatible with the large proportion of R337H carriers. This was demonstrated using two datasets, one from the 2006 cohort and the second from an independent ACT public registry. 

The LR shown in Figure 2 highlights the differences in the frequencies of R337H in the populations and in the ACTs between states. Considering that these three states (PR, SC, and SP) may correspond to the highest R337H frequencies, which drop progressively in the more distant municipalities of other states, LR provides additional information about burden of disease associated, which may change with diverse scenarios (e.g., change of environments). However, there are limitations which include the large range of the 95% confidence interval (uncertainty indicated by shading), and most of the studies are not sufficiently powered to demonstrate this population vs. tumor genotype frequency and the environmental interference. However, this study presents a framework for investigating this relationship in larger populations. Therefore, future studies are necessary to investigate interferences from different environment factors, genetic, and epigenetic variants.

The complexity of ACT development, including the presence of environmental modifiers, is not clearly understood, and there are important caveats to be further explored in our study. It is clear that the adrenocortical tissue is highly susceptible to most *TP53* mutations, and one of the reasons for this may be the adrenal cortex physiological transition across birth (reviewed by [13]). Since some families presented two or more cases of ACT in the 2006 cohort, 71 of them (20%) had 81 cases of ACT (95% were children diagnosed at age <10 years) originating from diverse rural and urban environments. We demonstrated a significant difference in the incidence using two different datasets, with a rate 2.7 times (IRR, Table 2) higher in C3 than in C1 (*p* = 0.0053). This phenotypic heterogeneity could be due to the elevated contamination levels of anti-ACT DDT/DDD/DDE previously measured in the rivers of C1 [18] and/or other ACT-promoting environmental pollutants in C3. 1-chloro-2-[2,2-dichloro-1-(4-chlorophenyl)ethyl]benzene (o,p′-DDD) (mitotane) was identified as a by-product of p,p′-DDD or p,p′-DDT and was reported as more potent in causing total inhibition of steroidogenesis in dogs than p,p′-DDD [31]. To date, mitotane remains the best adjuvant therapy for advanced stages of ACT in adults and children [32,33]. The adrenolytic effect of mitotane is inferior to those of the organochlorines p,p′-DDT, p,p′-DDE, and p,p′-DDD [34,35], which are very lipophilic and accumulate in fat tissues of animals consumed by humans [36,37].

ACT diagnoses based on hormonal and imaging preclinical exams (PE) allowed us to detect pre-clinical ACT [3]. Very small ACTs were confirmed by hormone testing and ultrasound to confirm sustained growth prior to deciding on better-resolution imaging analyses for surgical planning. Because of the possibility of high surveillance costs associated with PE, family transportation difficulties, and other factors, we designed another surveillance protocol for the 2016 cohort (2016-C) for clinical diagnosis based on early manifestations reported by trained, pre-counseled, and supportive parents. Considering the surveillance without PE, the odds of delayed ACT diagnosis are increased for the ~10% of tumors that do not produce excess steroids [14]; this could represent a loss of one ACT case per year in PR (1/17, considering carriers and non-carriers). Despite the small number of documented ACT cases (N = 1) identified without using PE in the 2016-C cohort to date (one stage I ACC found during a mean follow-up period of 2.1 years among 112 carrier newborns), we predict that it is cost-effective to replace PE with three sessions of training with psychological support for pre-counseled parents. Monitoring of carrier children by telephone through contact with parents has the potential to successfully detect early stage I pediatric ACT. 

Pediatric ACT is the fourth most common cancer in our 2006 cohort (after breast, gastric, and intestine). The recalculated average ACT penetrance changed slightly from the ages of five years (3.1%) to 12 years (3.9%), and this age range represented ~95% of all pediatric ACT cases (based on our present study data). The age at diagnosis of ACT during the first decade follows a similar age distribution to that reported for LFS [38]. PR is a territory with the highest worldwide frequency of R337H carriers, the highest pediatric ACT incidence, and very diverse environment conditions, ranging from a highly agricultural region to industrial areas and large urban regions [18]. Remarkably, C3 is a subregion where industries converge with large urban areas, and this region demonstrated a higher RR of developing pediatric ACT (adjusted for the number of R337H carriers) than C1 with the highest agriculture productivity. We have identified in average one case of pediatric ACT for every 54 R337H carriers in PR (77:4165; ACT:population), ranging from 1:26 in C3 to 1:93 in C1. However, the p.R337H in the adrenal cortex could unfold and lose function in carriers living in any subregion [38]. Furthermore, while it is unclear how hostile the environment is in C3, it is possible that environmental factors in this region could facilitate a second mutational hit with loss of the wild-type allele [39]. We speculate that low-penetrance mutations in tumor suppressor genes may be altered by specific environmental modifiers for each type of cancer (e.g., smoking for lung cancer or hepatitis B/C virus for liver cancer). 

The R337H frequencies across subregions of SC would predict an ACT incidence rate very similar to that in PR. However, since a lower burden of pollutants from agriculture and industry would be expected in SC due to the environmental differences compared with PR, further studies are needed to evaluate the SC environment. Given the frequency of other *TP53* germline mutations in other countries of between 1:5000 and 1:20,000 births [12,40] and 80% frequency of low-penetrance germline *TP53* mutations identified in pediatric ACT as reported by the Manchester Children’s Tumor Registry [6], it is important to consider the influence of environmental factors on the development of ACT in R337H carriers. Remarkably, the average age of ACT diagnosis dropped significantly in the last several generations of the 2006-C cohort, in all subregions and among carriers as well as non-carriers. This is likely related to the suboptimal medical care and delayed diagnosis in previous decades, as well as other unknown reasons. However, it is not yet possible to rule out anticipation in R337H carriers, nor can we rule out a stronger burden of environmental pollutants in the last several decades leading to an increased number of somatic variants causing different cancer types [40].

## 4. Materials and Methods

### 4.1. Genotyping of Newborns for TP53 R337H and Their Cohorts

Two neonatal screenings with two different surveillance protocols in Paraná state were used. The first followed periodical medical, hormonal, and ultrasound analyses aiming at pre-clinical or clinical diagnosis [3]. The second abolished all of the periodical exams (PE) but expanded the genetic and psychological counseling; this included three consultations and trainings sections on clinical manifestations of ACT and other cancers, at two-month intervals after birth. Furthermore, all families adhering to this training were periodically contacted by telephone and other methods of communication. The neonatal screening in Santa Catarina was conducted according to the Santa Catarina Ethics Committee recommendations (necessary use of stored blood from Guthrie’s tests, without establishing contacts with the families and parents of the positive newborns). 

A second neonatal screening was performed in PR to compare differences in ACT incidence among the PR subregions. R337H carrier frequencies obtained from these newly tested newborns as well as more accurate R337H frequencies obtained from 22 Administrative Health Regions (AHRs) gave rise to a second PR cohort (2016-C) under a surveillance protocol without PE to be compared with the cohort with PE (2006-C) established in 2006 [3]. In this second cohort without PE, pre-counseled parents received specific instructions and remote monitoring through periodic telephone communications about ACT clinical manifestations.

Research participant protection has been evaluated by five different Ethics Committees (ECs) over the last 15 years. Follow-up of families identified from the first neonatal screening in PR, which began in 2006, was approved by the ECs of Hospital de Clínicas at Federal University of Paraná (UFPR) and Pequeno Príncipe Hospital (CAA: 0023.0.208.000-05 from 2005, CAAE 0612.0.015.000-08, 2009) as a retrospective and prospective cohort initiated in 2006 [3]. The second PR neonatal screening was approved by the Pequeno Príncipe Hospital EC (CAAE: 50622315.0.0000.0097, 2015). Blood samples for this screening were obtained after parents signed the consent form at the Paraná State maternity ward or hospital (42,438 newborn blood samples collected during their Guthrie test) after pre-counseling on the second day after birth (2015 and 2016). Other NB blood samples were not included in our analyses because they were not from PR, the mothers were not located, or they did not sign the consent form.

This observational cohort followed the Strobe protocol framework [41] and was registered in the REBEC clinical trial (Registro Brasileiro de Ensaios Clínicos, www.ensaiosclinicos.gov.br), which provided psychological support, genetic counseling, DNA testing, and referral to specialists for treatment of cancer.

Neonatal screening in SC was performed to compare the R337H frequency in 16 SC AHRs. This was approved by the EC without access to families by the SC Health Secretary State EC, Florianópolis, state of Santa Catarina (CAAE: 73057817.5.0000.0115, 2017). The tests on 50,115 stored Guthrie cards were collected by the Central Laboratory (LACEN) of SC two years before the assays. Permission to screen stored newborn blood previously used for Guthrie tests in SC was granted along with the name of the newborn’s municipality. 

### 4.2. TP53 R337H Assay

A PCR-restriction fragment length polymorphism (PCR-RFLP) assay was used to detect the *TP53* R337H mutation as described previously [42]. Briefly, DNA was extracted from paraffin-embedded ACT and 3 mm punches from each filter paper-stored blood sample from Guthrie tests and families and assayed in 96-well plates. Blood and ACT samples from patients admitted to Joana de Gusmão Children’s Hospital (HIJG) from 2000 to 2018 (N = 35) were approved to be tested for the *TP53* R337H mutation by the hospital Ethics Committee (HIJG-2014). All samples that tested positive for R337H were confirmed by DNA sequencing.

### 4.3. ACT Cases from the Cohorts and Public Registry Used to Calculate Incidence

ACT cases identified in the 2006-C cohort were grouped into subregions of Paraná, C1, C2, and C3, in which spatial trends in human congenital malformations and chemical pollutions were confirmed, particularly in C1, which is predominated by very intense agriculture and higher levels of DDT/DDD/DDE [18]. The subregion with intermediary agriculture and industry activities was C2. Families were aggregated according to the generation and the subregion in which they lived when they developed ACT. The ACT incidence rate was calculated for children younger than 10 years.

Data from children younger than 10 years diagnosed with ACT and admitted to public hospitals from 2007 to 2018 in PR (N = 118) and from 2007 to 2017 in SC (N = 39) were obtained from the DATASUS Registry [23]. These cases were matched with ACT registries in the five main public hospitals in PR (~85%) and the main hospital in SC (~65% of the total) using a combination of three parameters for each patient (date of birth, place of birth, and date admitted to the hospital). The differences between the DATASUS registry and the cases admitted to the main hospitals in PR (98/118) and SC (28/39) could be explained by patients admitted to other public hospitals. The mean state population sizes for children younger than 10 years was calculated using registries for live births and child deaths from Parana Institute of Social and Economic Development (IPARDES, http://www.ipardes.pr.gov.br/imp/index.php) and from SC (DATASUS, http://tabnet.datasus.gov.br/cgi/tabcgi.exe?sinasc/cnv/nvsc.def and http://tabnet.datasus.gov.br/cgi/deftohtm.exe?sim/cnv/obt10sc.def).

### 4.4. LR for R337H Frequencies in Three Brazilian States

LR was calculated by the method of weighted least squares using two datasets: the R337H frequencies in the populations of PR, SC, and SP and the respective averages for R337H frequencies in ACT cases in each state. The data from PR and SC came from newborn testing and testing in ACT cases in the present study, as well as other PR data previously reported [3,9,20,30,31,32,33]. 

### 4.5. TP53 R337H Penetrance in 2006-C Cohort

Pediatric ACT penetrance in Paraná state was recalculated by adding newborn R337H carriers registered in the 2006-C cohort who developed new cases of ACT after 2012 [3]. The cumulative age-specific penetrance of ACTs was calculated by computing the linearly interpolated cumulative incidence. From the analyzed cohort, the families were grouped together and allocated by generations II, III, and IV or more (G-II, G-III, and G-IV+) using a non-parametric Kaplan–Meier estimator to estimate the age at ACT diagnosis per generation. 

### 4.6. Statistical Analysis

The R language program [43] was used for all statistical analyses. The chi-square and Fisher’s exact tests were used to evaluate the relationship dependence between categorical variables. The non-parametric Kaplan–Meier estimator was used to estimate the time of ACT occurrence, and the log rank test was used to compare two or more survival curves. ACT-free time was calculated from birth to ACT diagnosis or to most recent follow-up. The cumulative age-specific penetrance of ACTs in children identified as carriers at birth was analyzed using the 2006-C data by the method of Kalbfleisch and Prentice [44]. Confidence intervals for incidence rate, incidence rate ratio, and comparison test statistics were performed by the exact Poisson method [45]. The relative risk (RR) was estimated using the 2006-C data (cumulative incidence), and the incidence rate ratio (IRR) was calculated using the public ACT data. The linear regression (LR) was estimated for the R337H frequency in the populations of three states with the respective R337H frequencies in their ACT cases.

## 5. Conclusions

Collectively, the results from these two states have created an opportunity for classifying pediatric ACT incidence on the basis of R337H frequencies and geographical differences. Importantly, environmental influences should be considered in cancer risk, especially for carriers of germline *TP53* mutations. A complete understanding of the variability among environmental conditions to which R337H and non-R337H carriers are exposed is hindered by the lack of measurement of exact components needed to characterize the factor(s) associated with the observed high ACT incidence in C3 and lower incidence in C1 subregions. Therefore, the next major advance for southern Brazil will be to characterize the environmental factors in C1, C3, and other regions. The best available option for Southern Brazil to increase the rate of ACT cure is early onset ACT diagnosis and surgical dissection without any other therapy, as previously demonstrated in the 2006 cohort [3], combined with the preferential use of the simple and cost-effective surveillance method used in the present study (the 2016 cohort).

## Figures and Tables

**Table 1:** Cumulative adrenocortical tumor (ACT) incidence among *TP53* R337H carriers, ACT cases, and relative risk (RR) per subregion based on the 2006-C data.

ACT and Carriers			Subregion					
			C1		C2	C3		Total
ACT cases <10 years of age			**7**		34	36		77
Carriers without ACT			730		1819	1536		4085
Total			737		1853	1572		4162
**ACT**								
**Subregion**	**N**	**C.I.**	**Subregion**	**N**	**C.I.**	**RR**	***p* Value**	
		**(/1000)**			**(/1000)**		***X*^2^**	**Fischer**
C2	34	18.3	C1	7	9.5	1.932 [0.86; 4.338]	0.146	0.118
C3	36	22.9	C2	34	18.3	1.248 [0.785; 1.985]	0.414	0.397
C3	36	22.9	C1	7	9.5	2.411 [1.078; 5.392]	0.04	0.031

C.I., cumulative incidence. Three ACT cases in adults were not included in this analysis.

**Table 2:** ACT incidence rate per subregion of Paraná state (**A**), and differences using Bonferroni adjusted *p* values (**B**) using the public databases (2007–2018).

**A**				
**Subregion**	**ACT Cases** **<10 Years (2007–2018)**	**Persons** **<10 Years (2007–2018)**	**Incidence** **(<10 Years) /Million/Year**	**95% CI**
C0	9	956,764	9.41	4.30; 17.86
C3	46	5,643,503	8.15	5.97; 10.87
C2	50	7,552,735	6.62	4.91; 8.73
C1	13	4,328,094	3.0	1.60; 5.14
**B**				
**Comparison**	**Incidence Rate Ratio (IRR)**	**95% CI**	**Adjusted *p* (Bonferroni)**	
C0/C3	1.154	0.497; 2.386	1.0000	
C0/C2	1.421	0.614; 2.919	1.0000	
C0/C1	3.132	1.182; 7.919	0.0638	
C3/C2	1.231	0.807; 1.875	1.0000	
C3/C1	2.714	1.442; 5.475	0.0053	
C2/C1	2.204	1.180; 4.423	0.0514	

**Table 3:** ACT cases among R337H carriers: three cases in the 2006-C cohort between 2012 and 2018 with exams (**A**), and one adrenocortical carcinoma (ACC) case in 2016-C without periodical exams (PE) (**B**).

**A**							
**Code**	Surveillance */Interval **	**Gender/ACC Age (Y)**	**Clinical Features**	**ACT Weight (g)**	**Stage/Treatment**	**Recurrence**	**Outcome/Present Age (Years)**
1	Regular/4 months	F/6.2	Acne	14	I/CR	No	Well/8.2
2	Irregular/9 months	M/7.3	Acne + pubic hair	267	II/CR + M	No	Well/11.4
3	Irregular/22 months	F/5.8	EnlargedClitoris, acne, pubic hair + H	NA	IV/PR + EDPM	Remained local tumor and metastases	DD/6.4
**B**							
**Code**	Surveillance */Interval **	**Gender/ACC Age (Y)**	**Clinical Features**	**ACT Weight (g)**	**Stage/Treatment**	**Recurrence**	**Outcome/Present Age (Years)**
1	Regular/No PE	F/0.9	Pubic hair	21	I/CR	No	Well/3.6

In part A: * Surveillance (regular consultations with hormonal and imaging exams: every six months between five and eight years of age); ** Consultation interval; CR, complete resection; DD, died of disease, NA, not available; PR, partial resection; H, high blood pressure; M, mitotane; EDPM (etoposide, doxorubicin, cisplatin + mitotane regime); Well, alive without signs of disease. In part B: * Surveillance (inquiry about signs and symptoms without any PE); ** (4/4 months); CR, complete resection.
